# Multi-objective data enhancement for deep learning-based ultrasound analysis

**DOI:** 10.1186/s12859-022-04985-4

**Published:** 2022-10-20

**Authors:** Chengkai Piao, Mengyue Lv, Shujie Wang, Rongyan Zhou, Yuchen Wang, Jinmao Wei, Jian Liu

**Affiliations:** 1 College of Computer Science, Nankai University, Tianjin, China; 2 Department of Ultrasound, Cangzhou Municipal Haixing Hospital, Cangzhou, China

**Keywords:** Multi-objective, Parameter sharing, Ultrasound analysis, Deep learning, Thyroid nodules

## Abstract

Recently, Deep Learning based automatic generation of treatment recommendation has been attracting much attention. However, medical datasets are usually small, which may lead to over-fitting and inferior performances of deep learning models. In this paper, we propose multi-objective data enhancement method to indirectly scale up the medical data to avoid over-fitting and generate high quantity treatment recommendations. Specifically, we define a main and several auxiliary tasks on the same dataset and train a specific model for each of these tasks to learn different aspects of knowledge in limited data scale. Meanwhile, a Soft Parameter Sharing method is exploited to share learned knowledge among models. By sharing the knowledge learned by auxiliary tasks to the main task, the proposed method can take different semantic distributions into account during the training process of the main task. We collected an ultrasound dataset of thyroid nodules that contains Findings, Impressions and Treatment Recommendations labeled by professional doctors. We conducted various experiments on the dataset to validate the proposed method and justified its better performance than existing methods.

## Introduction

Ultrasound Analysis, as one of the most commonly used examination methods, has been recognized as a powerful screening and diagnostic tool for physicians and radiologists [1, 2]. Recently, Deep Learning (DL) based Automatic Ultrasound Analysis (AUA) [3] methods, such as Disease Screening (DS) [4–6], Lesion Detection (LD) [7–9], Automatic Diagnosis (AG) [10–12], etc, have attracted attention from academics and practitioners [13]. As a powerful auxiliary tool to analyze the conditions of patients, AUA methods can help doctors to reduce their workloads.

In applications, the demands of medical workers for AUA also include Automatic Treatment Recommendation (ATR), a less studied field that automatically generates treatment recommendations without the intervention of doctors. Researchers proposed many DL models to address ATR tasks, which can be used to replace the doctor’s work to some extent [14–19]. These models can be categorized into internal and external methods. Internal methods adopted elaborately designed backbone structures to extract informative features. By virtue of powerful structures and skillful training tricks, such as deep structures, residual connections, dropout layers, etc, internal methods could extract representative features and soon achieved the dominant position [20–23]. External methods adopted a shared training policy that pre-trains the model on public datasets firstly, and then fine-tune its parameters on the ultrasound dataset. With the help of public knowledge, external methods can ensure the model to be sufficiently trained [24–27].

However, there are two limitations in existing methods, insufficient training data and mismatched knowledge fields [28]. On the one hand, the main way to improve the performances of internal methods is to design more complex structures and use larger training sets. But it is difficult to acquire labeled large-scale medical datasets due to the high labeling costs, which will constrain the performances of internal models. On the other hand, ultrasound data are full of professional knowledge and may not have the same semantic distribution with pre-training datasets. Furthermore, how to build a highly related pre-training dataset for the ATR task is also a problem.

To address the aforementioned limitations, we propose the Multi-Objective Data Enhancement (MODE) method, which is capable of expanding the limited dataset without extra labeling costs and external datasets. In addition, we present an ultrasound dataset of thyroid nodules, which contains not only Findings and Impressions, the results that average ultrasound reports should have, but also Treatment Recommendations and Severity Scores labeled by clinicians, to validate the feasibility of MODE.

Specifically, we define a main task and two auxiliary tasks on the ultrasound dataset, each task has its own training objective and model. The main task is the ATR task that generates Treatment Recommendations according to Findings and Impressions, we construct a Transformer model to handle this task. The First auxiliary task is a Summary task that generates Impressions for given Findings, we construct a Long-Short term Memory (LSTM) model to handle this task. The second auxiliary task is a Regression task that computes Severity Scores according to Findings and Impressions, we construct a Convolution Neural Network (CNN) model to handle this task. Meanwhile, we present the Soft Parameter Sharing (SPS) method specially for sharing the learned knowledge from the two completely different types of auxiliary tasks to the main task. In the training process, we first train the two auxiliary tasks to learn different aspects of the ultrasound data, and then train the main model with the learned auxiliary knowledge as prior information.

**Fig. 1:**
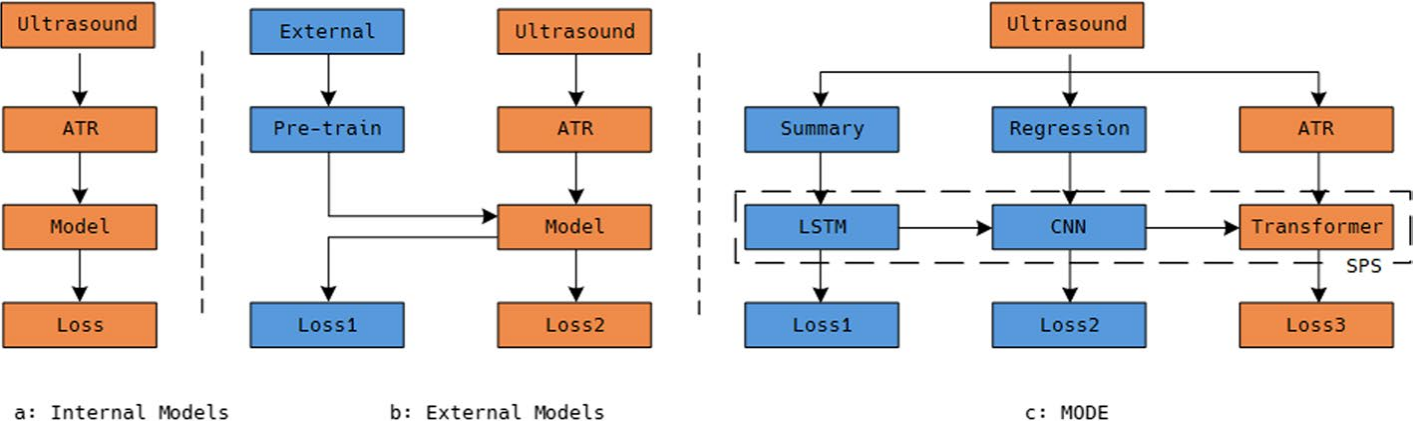
The comparisons between MODE and existing methods, in which orange blocks denote the main tasks and blue blocks are auxiliary tasks

With the presented ultrasound dataset as an example, Fig. 1 illustrates the comparison between MODE and existing methods. In Fig. 1a, an internal method directly trains the model to generate the Treatment Recommendations, which may suffer from the limitation of insufficient training samples. In Fig. 1b, an external method pre-trains the model on public datasets to avoid over-fitting, but the learned common knowledge may not be compatible with the ultrasound dataset. In Fig. 1c, we define multiple tasks on the same dataset and share knowledge among models. For the first limitation, the multiple training objectives can force the models to learn different aspects of knowledge from the same dataset, and to indirectly increase the training samples. For the second limitation, the parameter sharing method can be used to share learned knowledge of auxiliary tasks to the main task, to function like external datasets. Consequently, the proposed framework can fully optimize their parameters within less training samples and little external knowledge.

The main contributions of this paper are listed below.

- A Multi-objective data enhancement method which can expanding the ultrasound dataset without extra labeling costs and external datasets.
- A Soft Parameter Sharing method used to share the learned knowledge among models.
- An ultrasound dataset of thyroid nodules, which contains Findings, Impressions, Treatment Recommendations and Severity Scores.

The rest of this paper is organized as follows. The second section briefly introduces the related work of this paper. In the third section, we introduce and analyze the details of MODE. In the fourth part, the method is applied to ultrasonic dataset, and the experimental results are analyzed in detail. Finally, we summarize the work of this paper in the fifth part.

## Related work

DL models have deeply influenced some areas of medical informatics, especially NLP-based tasks [29]. Researchers proposed many DL models to handle different kinds of medical tasks, such as Automatic Diagnosis, Disease Screening, Lesion Detection (LD), etc. ATR is a less studied area that needs both elaborately designed structures and enough training samples. According to the demand of input–output formats and model structures, two kinds of methods, viz. internal and external methods, can be used to solve ATR problems.

Complying with the former category, some researchers held the opinion that extracting abundant and high-quality semantic features are key factors [30], thus they have worked on representation models for a long time and proposed many elaborately designed structures and training tricks [31]. Specifically, [32] proposed an LSTM based model to identify text order of medical data. Borjali et al. [33] proposed a DL-NLP model for efficient and accurate hip dislocation medical adverse events detection. Liu et al. [34] proposed to use hierarchical CNN and LSTM models to handle negations and numerical values that exist in medical text. Prabhakar et al. [35] proposed to use quad-channel features to enhance the performance of LSTM.

In the latter domain, knowledge distribution is regarded as the engine room of DL models, and it will be more solid with a larger scale dataset. Following this idea, researchers used extra datasets to pre-train the model and to expand its knowledge field. Rebane et al. [36] used large scale diagnosis and drug records as the external dataset to instruct the process of medical knowledge extraction. Qin et al. [37] proposed an orthogonal wrapper to enhance the differences between datasets and thus to extract distinguishable and informative features. To take full advantage of external knowledge, a set of super-large scale Internet data was pre-trained firstly to learn word embeddings, and then a fine-tuning stage is adopted to take advantage of these representation vectors to fit specific data [38, 39].

Generally, deep and complex structures have stronger feature-selecting abilities. But in medical related tasks, insufficient training samples may be an obstacle for internal models to understanding the professional knowledge, and lead to over-fitting. Using external dataset to pre-train the model is an intuitive method to address the over-fitting problem since it can provide abundant training samples. However, it may lead to mismatched semantic distributions if we use non-related common datasets in the pre-training processes.

We present a data enhancing method, MODE, to scale up the ultrasound dataset without extra labeling costs. We define a main task and two auxiliary tasks on the ultrasound dataset, each task has its own training objective and model. According to the distinctive training objectives, auxiliary models can learn different aspects of semantic distribution. For example, Findings are descriptions of examination results, Impressions are corresponding conclusions. The Summary task is then defined to help the model learn the relations between lesions and clinical symptoms. The Regression task is trained to select the most related factor to identify the disease. With the help of the SPS method, the learned knowledge of auxiliary models corresponding to different aspects of ultrasound analysis can be concentrated to the main task, to indirectly scale up the dataset and provide solid semantic distribution. Consequently, MODE is capable of enlarging the scale of professional datasets with the same domain instances.

## Multi-objective data enhancement

We introduce the structure of MODE as well as the main and auxiliary tasks in “The structure of MODE”–“Using transformer to handle the ATR task” sections. We propose the SPS method in “Soft parameter sharing” section, which is used to share the learned knowledge of auxiliary tasks to the main task. Finally we present the training process of MODE in “Training process” section.

### The structure of MODE

The main idea of MODE is to reuse limited dataset by defining multiple tasks. Since all training objectives are defined on the ultrasound dataset, the MODE does not use any external data to train the models. Specifically, we define a main and two auxiliary tasks on the proposed ultrasound dataset, each of which has its own training objective. The main task, ATR, is a generation task that transforms Finding and Impression into Treatment Recommendation. The first auxiliary task is a Summary task that transforms Finding into Impression, the second auxiliary task is a Regression task that computes the Severity Score according to Finding and Impression. By taking the learned knowledge of auxiliary tasks into account, the main task can learn different aspect of knowledge and achieve better performance.

**Fig. 2:**
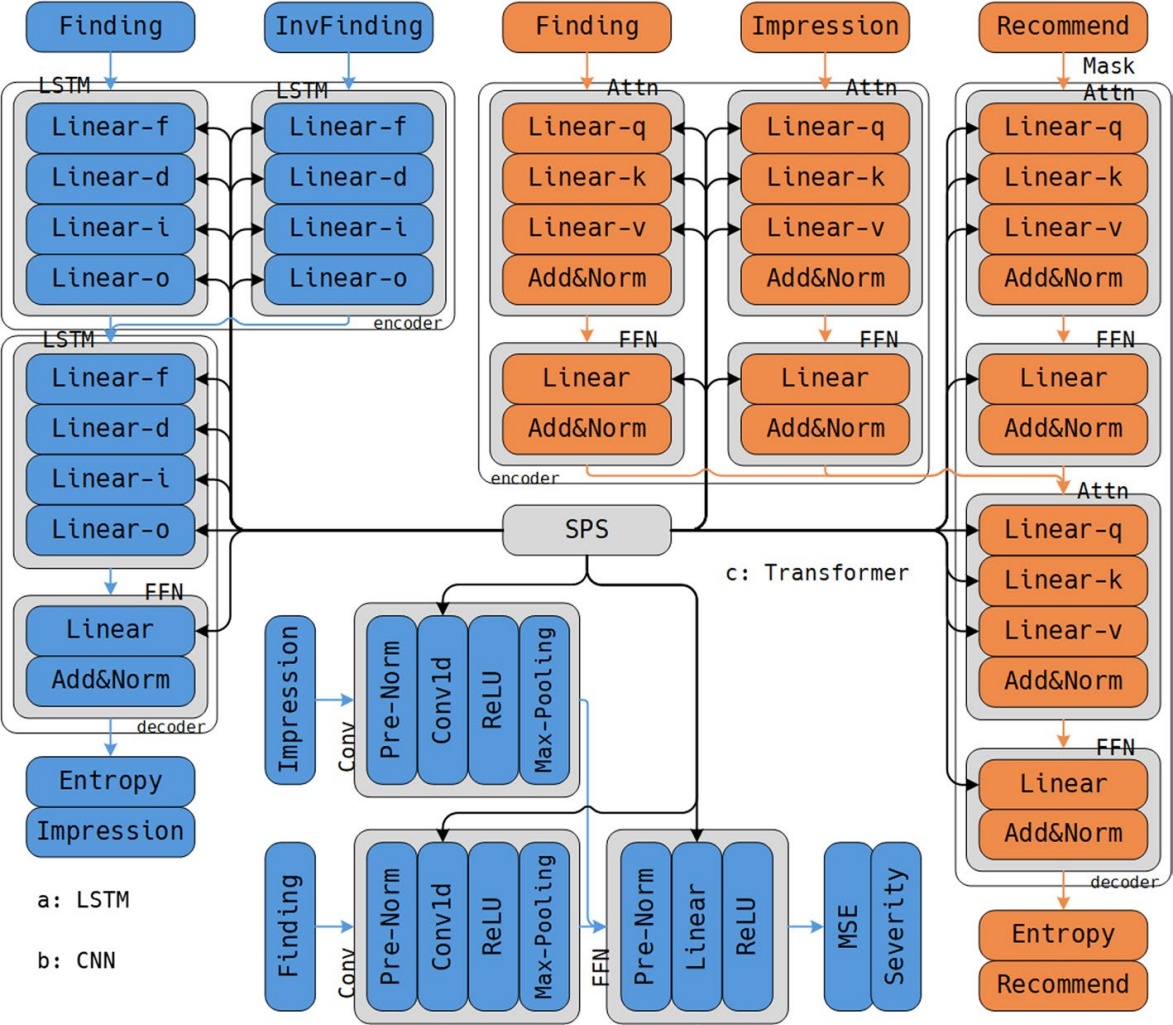
The structure of MODE

We train a specific DL model for each task respectively and propose the SPS method to share knowledge among models. Figure 2 illustrates the topology of MODE, in which orange and blue blocks, as well as corresponding lines, represent the main and auxiliary tasks. As shown in Fig. 2a, we use an encoder–decoder LSTM to handle the Summary task since it can satisfy the input–output data format that converts Findings into Impressions. As shown in Fig. 2b, we use a CNN model to handle Severity task since it can satisfy the input–output data format transformation that converts Findings and Impressions into Severity Scores. As shown in Fig. 2c, we use an encoder–decoder Transformer to handle ATR task since it can satisfy the input–output data format transformation that converts Findings and Impressions into Treatment Recommendations.

### Using long-short term memory to handle the summary task

Since LSTM is a powerful model to detect the sequential information, we use a Bi-direction LSTM model to handle the Summary task. Equation (1) illustrates the computing process of the Summary task.

1 $$\begin{aligned} {\varvec{Z}}&= [{\textit{LSTM}}({\varvec{F}}), {\textit{LSTM}}(inv({\varvec{F}}))]_c, \\ {\varvec{I}}&= {\textit{FFN}}({\textit{LSTM}}({\varvec{z}})). \end{aligned}$$

where ${\varvec{F}} \in {\mathbb {R}}^{|F| \times n}$ is the Finding text, $inv({\varvec{F}})$ denotes the inverse order of ${\varvec{F}}$, ${\varvec{Z}}$ is the intermediate variable, ${\varvec{I}} \in {\mathbb {R}}^{|I| \times n}$ is the Impression text, “$[*]_c$” denotes the concatenate operation in the channel dimension, |*F*| and |*I*| denote text lengths.

An LSTM module adopts recurrent steps to iteratively load and save historical information to update its hidden states. Equation (2) illustrates the updating process of LSTM.

2 $$\begin{aligned} {\varvec{c}}^t, {\varvec{h}}^t = {\textit{LSTM}}({\varvec{c}}^{t-1}, {\varvec{h}}^{t-1}, {\varvec{X}}_t). \end{aligned}$$

where ${\varvec{h}}$ and ${\varvec{c}} \in {\mathbb {R}}^{n}$ are hidden and cell states, superscripts denote the recurrent index, ${\varvec{X}}_t \in {\mathbb {R}}^{n}$ is the target word of the *t* th recurrent step.

In the recurrent process, LSTM trains a group of gates to control what information of ${\varvec{X}}_t$ should be add into ${\varvec{c}}^t$ and ${\varvec{h}}^t$. Equation (3) illustrates the definitions of gates and the states updating process.

3 $$\begin{aligned} {\varvec{g}}^t_f&= {\varvec{X}}_t \times {\varvec{W}}_f + {\varvec{h}}^{t-1} \times {\varvec{U}}_f + {\varvec{b}}_f, \\ {\varvec{g}}^t_d&= {\varvec{X}}_t \times {\varvec{W}}_d + {\varvec{h}}^{t-1} \times {\varvec{U}}_d + {\varvec{b}}_d, \\ {\varvec{g}}^t_i&= {\varvec{X}}_t \times {\varvec{W}}_i + {\varvec{h}}^{t-1} \times {\varvec{U}}_i + {\varvec{b}}_i, \\ {\varvec{g}}^t_o&= {\varvec{X}}_t \times {\varvec{W}}_o + {\varvec{h}}^{t-1} \times {\varvec{U}}_o + {\varvec{b}}_o, \\ {\varvec{c}}^t&= {\varvec{c}}^{t-1} \cdot {\varvec{g}}^t_f + {\varvec{g}}^t_d \cdot {\varvec{g}}^t_i, \\ {\varvec{h}}^t&= {\varvec{g}}^t_o \cdot tanh({\varvec{h}}^t). \end{aligned}$$

where ${\varvec{g}}^t_f, {\varvec{g}}^t_d, {\varvec{g}}^t_i, {\varvec{g}}^t_o \in {\mathbb {R}}^{n}$ are LSTM gates, ${\varvec{W}}_*, {\varvec{U}}_* \in {\mathbb {R}}^{n, n}$ are trainable parameters, ${\varvec{b}}_* \in {\mathbb {R}}^{n}$ are bias, $\times$ denotes matrix multiplication, “$\cdot$” denotes Hadamard Product.

Although an LSTM model has such a complex computing process, each of its gates can be viewed as the combination of two linear modules. Equation (4) provides an equivalent implementation of an LSTM gate in Eq. (3).

4 $$\begin{aligned} {\varvec{g}}^t = {\textit{Linear}}_W({\varvec{x}}^t) + {\textit{Linear}}_U({\varvec{x}}^t). \end{aligned}$$

In “Soft parameter sharing” section, we propose the SPS method to share the learned knowledge of the LSTM model to the main task.

### Using convolution neural network to handle the severity task

CNN is a powerful model which is good at transforming sequential data into a single value, which can be used to handle the Severity task. We use two CNN modules to extract important features in Findings and Impressions respectively. Equation (5) illustrates the computing process of CNN.

5 $$\begin{aligned} s = {\textit{FFN}}([{\textit{Conv1d}}({\varvec{F}}), {\textit{Conv1d}}({\varvec{I}})]_t). \end{aligned}$$

where *s* is the Severity Score of the ultrasound report, “$[*]_t$” denotes the concatenation operation in the temporal dimension.

Obviously, a CNN model mainly contains convolution and linear modules. In “Soft parameter sharing” section, we propose the SPS method to share the learned knowledge of the CNN model to the main task.

### Using transformer to handle the ATR task

We use a Transformer model to generate Treatment Recommendation from Finding and Impression since it is good at searching relations among sequences. Equation (6) illustrates the computing process of Transformer.

6 $$\begin{aligned} {\varvec{Z}}&= [{\textit{FFN}}({\textit{SelfAttn}}({\varvec{F}})), {\textit{FFN}}({\textit{SelfAttn}}({\varvec{I}}))]_t, \\ {\varvec{T}}&= {\textit{FFN}}({\textit{Attn}}({\textit{FFN}}({\textit{MaskAttn}}({\varvec{T}})), {\varvec{Z}}, {\varvec{Z}})). \end{aligned}$$

where ${\textit{SelfAttn}}({\varvec{F}}) = {\textit{Attn}}({\varvec{F}}, {\varvec{F}}, {\varvec{F}})$ is a special version of Attention module, ${\varvec{Z}}$ is intermediate variable, ${\textit{MaskAttn}}()$ has the same structure of ${\textit{SelfAttn}}$ except it uses a mask attention matrix to prevent the module from seeing future words, ${\varvec{T}}$ is Treatment Recommendation. Equation (7) illiterates the definition of *Attn*.

7 $$\begin{aligned} {\textit{Attn}}({\varvec{Q}}, {\varvec{K}}, {\varvec{V}}) = {\textit{softmax}}(\frac{{\varvec{Q}} {\varvec{K}}^{\mathbb {T}}}{\sqrt{n}}) \times {\varvec{V}}. \end{aligned}$$

Similar to LSTM and CNN, a Transformer model is mainly controlled by three linear modules. Equation (8) illustrates the relation between linear module and the Attention.

8 $$\begin{aligned} {\varvec{Q}}&= {\textit{Linear}}_Q({\varvec{X}}), \\ {\varvec{K}}&= {\textit{Linear}}_K({\varvec{X}}), \\ {\varvec{V}}&= {\textit{Linear}}_V({\varvec{X}}). \end{aligned}$$

where ${\varvec{X}}$ is input data.

In “Soft parameter sharing” section, we propose the SPS method to share the learned knowledge of the Transformer model to the main task.

### Soft parameter sharing

To enable the main task to take different aspects of the ultrasound dataset in to account, we need a cross-model parameter sharing method to share the learned knowledge of auxiliary tasks to the main task. As we discussed earlier, although models have different structures, their fundamental bricks are CNN and Linear blocks. Considering that CNN and Linear blocks incorporate fixed size matrices as their parameters, it is plausible to indirectly share knowledge across models by letting all modules use the same parameter matrix. However, it is difficult to use a fixed size matrix as their parameters since the models have different parameter shapes, a CNN model needs 3-dimension matrices as its kernels, but a Linear model usually uses 2 dimension parameter matrices. To address this problem, we propose the Soft Parameter Sharing (SPS) method, which is capable of transforming the global shared parameter matrix into different parameter matrices.

SPS is a CNN based algorithm that utilizes the unsymmetrical character of convolution operations to tailor matrix into specific shapes. First, we define a parameter matrix, called template, as the global parameters. Then, we assign a SPS kernel to each CNN, or Linear, module. Last, we compute the shared parameters through Eq. (9), and use this parameter matrix to replace the original parameters.

9 $$\begin{aligned} {\textit{SPS}}({\varvec{M}}) = \sigma ({\varvec{E}} \otimes {\varvec{M}} + {\varvec{b}}). \end{aligned}$$

where ${\varvec{E}}$ is the global template, ${\varvec{M}}$ is the SPS kernel used to control the output size, ${\varvec{b}}$ is bias.

**Fig. 3:**
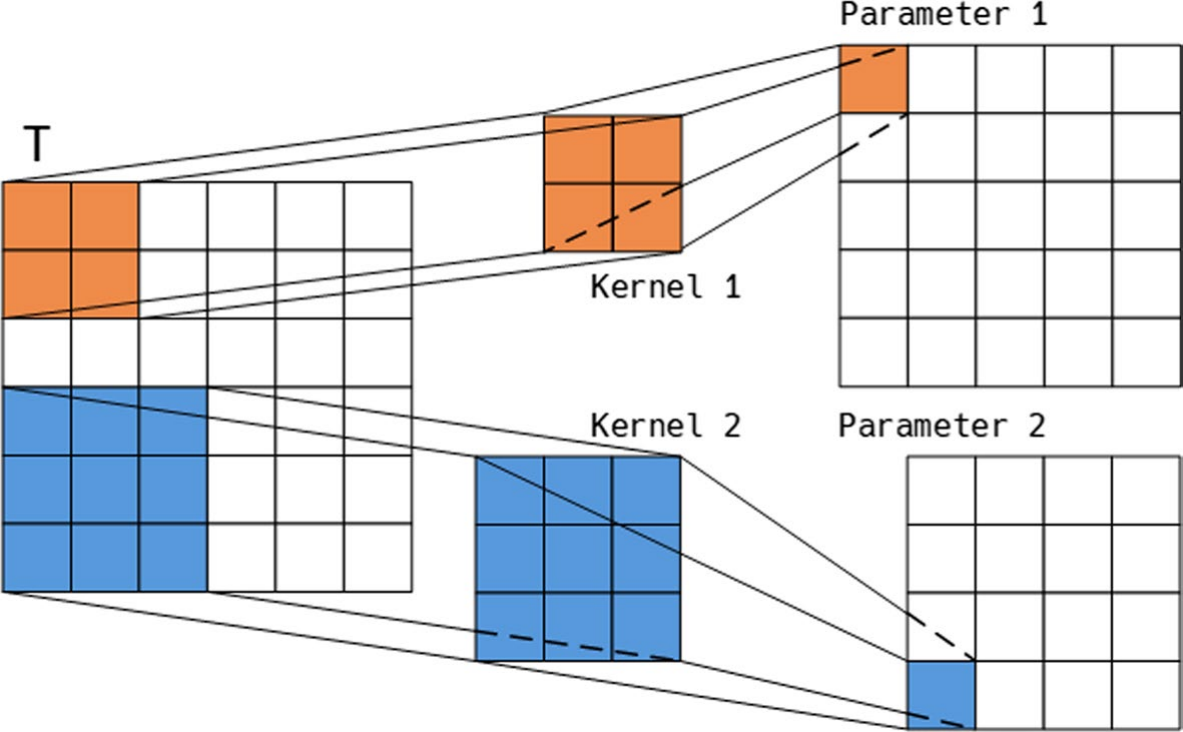
The progress of soft parameter sharing

As shown in Fig. 3, the shape of shared parameter matrix can thus be revised by simply adjusting the SPS kernel. For example, given the source matrix ${\varvec{E}} \in {\mathbb {R}}^{r \times c \times p}$ and a target matrix ${\varvec{W}} \in {\mathbb {R}}^{r_0 \times c_0 \times p_0}$, we simply need to set the shape of ${\varvec{M}}$ with $[ r - r_0 + 1, c - c_0 + 1, p_0, p ]$.

With the SPS method equipped, the CNN modules in Eq. (5) can be replaced by Eq. (10), the linear modules in Eqs. (4) and (8) can be replaced by Eq. (11).

10 $$\begin{aligned} {\textit{Conv1D}}({\varvec{X}})= {\varvec{X}} \otimes {\varvec{W}}_c + {\varvec{b}}_c, {\varvec{W}}_c = {\textit{SPS}}({\varvec{M}}_c). \end{aligned}$$

11 $$\begin{aligned} {\textit{Linear}}({\varvec{X}})= {\varvec{X}} \times {\varvec{W}}_l + {\varvec{b}}_l, {\varvec{W}}_l = {\textit{SPS}}({\varvec{M}}_l). \end{aligned}$$

where ${\varvec{X}}$ denotes the input data, ${\varvec{W}}_c \in {\mathbb {R}}^{n \times n \times k}$ is the kernel of convolution module, *k* denotes the kernel size, ${\varvec{W}}_l \in {\mathbb {R}}^{n \times n}$ is the parameter matrix of linear module. ${\varvec{b}}_*$ are bias, $\otimes$ denotes convolution operation.

The SPS method only replace the trainable parameters of CNN and Linear modules with the global template, their structures and training processes are unchanged. Therefore, the SPS method can share knowledge among models while maintain the structural advantage of the original model.

### Training process

**Fig. 4:**
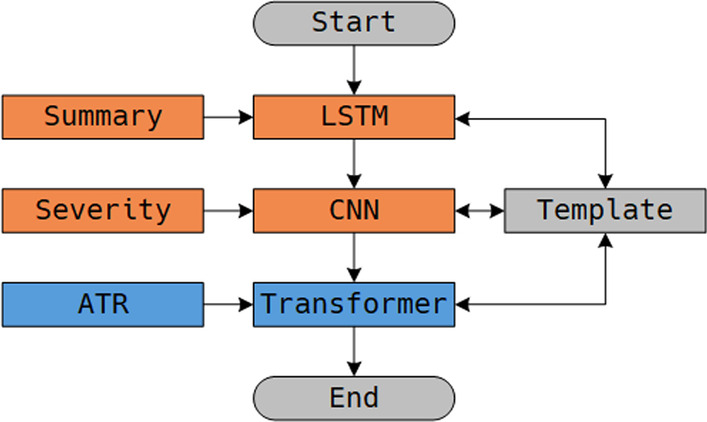
The training process of MODE

In the training process, we set a specific training order to ensure the main task can take different aspects of the dataset knowledge into account. Specifically, we first train the auxiliary tasks to store the learned knowledge into the global template, and then we use this informative template as the initial parameter matrix of the ATR task to share knowledge. Figure 4 illustrates the training order of MODE.

At the very first, the template is initialized with random values. Then, the template will learn different aspects of knowledge by driving the model to train auxiliary tasks. Last, the ATR task can inherit the learned knowledge of auxiliary tasks by using the same template as the initial parameters.

For each task, we use the SPS model to compute the shared parameter matrices and to replace the original parameters of the model. Meanwhile, we use forward–backward steps to compute the gradients and update the template. With the Summary task as an example, Algorithm 1 illustrates the training process of a task.
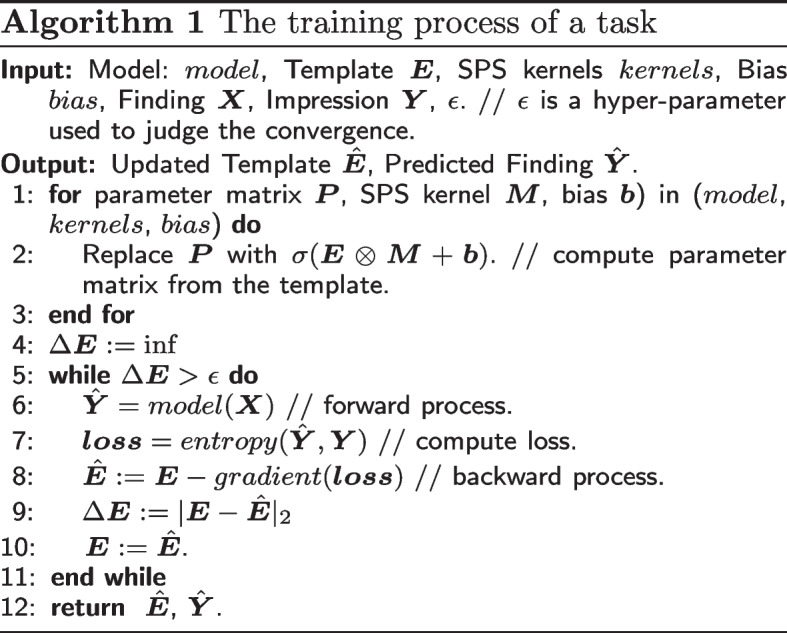


## Experiment

In this section our goal is to showcase benefits of modeling multiple objectives in the same medical dataset, for which we define three tasks. We begin with two auxiliary tasks of Impression-generating and Severity-computing to learn different knowledge aspects from the dataset. Then, we define a main task of ATR. Finally, we train a specific model for each task and share the parameters of auxiliary tasks to the main task through SPS for which capability to share parameters would allow us to indirectly add more training samples to the limited dataset.

### Dataset

We collect a group of 513 ultrasound reports from clinical patients, each report has two sequences of texts, Findings and Impressions. In addition, each report is labeled with a sequence of Treatment Recommendation and a severity score by a group of professional doctors, at least deputy chief physicians. We extract the sentences and corresponding labels from the unprocessed data. The only pre-processing operation of these sentences is to tokenize them into character level. The dataset is partitioned randomly into training set (70%), development set (10%) and test set (20%). The statistic of the dataset is listed in Table 1.

**Table 1:** Statistics of the ultrasound dataset

Item	Type	Avg. len	Token	Label
Finding	Sequence	60	353	–
Impression	Sequence	31	263	–
Treatment	Sequence	53	277	–
Severity	Value	–	–	6

We define three tasks on the Ultrasound dataset. Specifically, Impression-generating is a summary task that generates Impression text for a given Finding. This task can alleviate the clinic doctors’ workload during the ultrasound inspections. Severity-computing is a regression, or classification, task that computes the severity of the disease. To emphasis MODE is compatible with multiple loss functions, the Severity-computing is viewed as a regression task. This task can help the doctor to identify the patient’s situation. ATR is a Question-Answering task that generates the Treatment Recommendation for an ultrasound report. This task can partly replace the doctor’s work and make contributions to Auto Inspections of Artificial Intelligence Medical.

### Competitor methods

Previous works have proposed various methods but not all of them can be applied to our tasks. We chose 4 most related sequence-to-sequence models for related tasks and implemented them as competitor methods.

- We used Medical LSTM [19] as the baseline of Internal LSTM. It is an alternative to the conventional sentiment analysis approaches in analysing large volumes of data in a potential flow.
- We used Memory-driven Transformer (MDT) [22] as the baseline of internal Transformer. It used a relational memory module and a memory-driven conditional layer normalization to record key information of the generation process of Transformer.
- We used Symptoms Frequency Position Attention (BiLSTM-SFPA) [20] as the baseline of external LSTM. It used adaptive weight assignment techniques and positional context to address APT task. Meanwhile, it used word2vec and the Chinese Ci-Lin as external knowledge.
- We used Bidirectional Transformer [26] as the baseline of external Transformer. It used Bidirectional-Transformer based architecture to generate encoded representations from external datasets firstly, and then use the learned knowledge to handle specific tasks.

### Hyper-parameters

We used random initialized vectors as word embeddings. All weights were randomly initialized by the Xavier Uniform Initializer. Dropout [40] rate, batch size and other hyper-parameters were set according to datasets and the memory capability. All texts in the same batch were padded to the same length. All models were optimized using the Adam optimizer [41]. Particularly, to select useful features, we used FP-net to extract features in an anti-gradient direction. Furthermore, Eq. (12) gives an epoch related learning rate updating policy with an initial learning rate of 0.01.

12 $$\begin{aligned} lr_{i+1}=0.8 * lr_{i} * 0.01^{\frac{i + 0.01}{{\textit{epoch}}_{{\textit{max}}} + 0.01}}. \end{aligned}$$

where *lr* denotes the learning rate, *i* is the current epoch index, ${\textit{epoch}}_{{\textit{max}}}$ is the max epoch, 40 in our experiments.

The experiments were conducted on a NVIDIA 3090 GPU with 24 GB memory and an Intel 10900x CPU with 64 GB memory.

### Training settings

Table 2 shows the MLLs with various training settings on the development set, where **Time** refers to training time per epoch, **Param** represents the number of trainable parameters of the model. In the **Preference** column, Summary and Severity denote using corresponding auxiliary tasks. Independent and Collaborate denote whether the auxiliary tasks are trained separately or integrated in the main task. In the former method, each task is trained one-by-one and the training order is Summary-Severity-ATR. In the latter method, auxiliary tasks and the main task are trained simultaneously, the training order in each epoch is the same as Independent’s and their losses are added up to perform backward steps. Flags denote adding start and end flags, $<{\textit{start}}>$ and $<{\textit{end}}>$, on a sequence. Since there are multiple training objectives, we use Minus Log Loss (MLL), ${\textit{MLL}}({\textit{loss}}) = -{\textit{log}}({\textit{loss}})$, to represent the performance. A better model will produce larger MLL score.

**Table 2:** Training settings of ATR task

Preference	Time (ms)	Dev. MLL	Param (M)
None	557	1.49	3.78
Summary	1380	1.51	3.97
Severity	602	1.54	3.93
Both	1390	1.57	4.12
Independent	557	1.56	3.78
Collaborate	1390	1.57	4.12
Non-flags	1390	1.57	4.12
Flags	1390	1.60	4.12

As shown in the table, the MLL of ATR task drops to 1.49 without the help of Summary task or the Severity task, demonstrating the necessity of auxiliary knowledge. With the help of two auxiliary tasks, both independent and collaborative training policy can improve the performance of the ATR task. Although the performances of two methods are similar, they have different advantages in applications. Specifically, the independent training policy utilizes auxiliary tasks as pure external knowledge. It is convenient to transfer this method to other tasks since adding models does not affect its structure. Collaborative training policy treats auxiliary tasks as not only external knowledge but additional training samples. Scaling up the dataset can fully train the model and decrease the risk of over-fitting. Consequently, the former method can be deployed in cross-domain areas while the latter one is more likely to perform well in disease-specific environments. Furthermore, without using start and end flags, the performance drops from 1.6 to 1.57, showing the effectiveness of having these additional nodes.

### Ablation study

#### Influence of model size

**Fig. 5:**
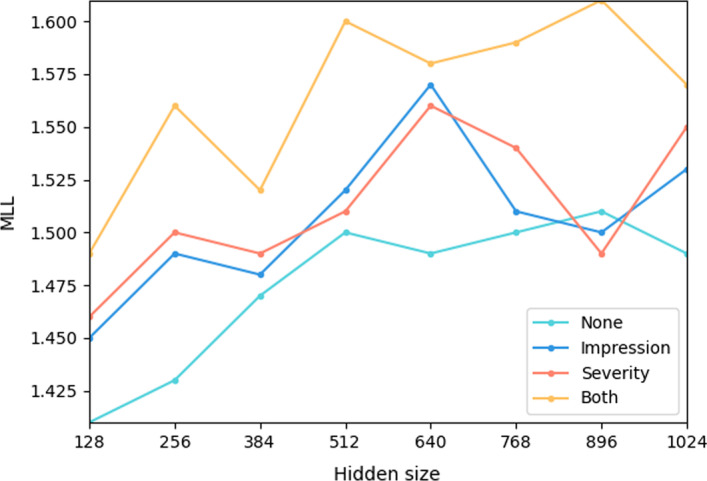
Comparisons of different hidden size

Figure 5 illustrates the MLLs with different hidden sizes and auxiliary tasks on the test set. “None” denotes the ATR task was trained independently, “Impression” and “Severity” denote corresponding tasks were used to share their knowledge to the ATR task, “Both” denotes ATR task was trained with the help of both auxiliary models. When the hidden size increased from 128 to 1024, all methods reported increasing performance trends, which is consistent with the fact that a larger model usually has better representative ability. The performances generally increased before reaching a peak value, and then reported a decrease trend, although larger hidden sizes are adopted, which is consistent with the fact that too large models won’t benefit the model. In comparison, few significant differences are observed over the peak performance with the help of auxiliary tasks. On the one hand, this shows reusing training samples can help the model to avoid over-fitting. On the other hand, this can be explained by the intuition that information exchange between auxiliary tasks and the main task can help the model to extract representative features and learn solid semantic distribution.

#### Influence of layers

**Fig. 6:**
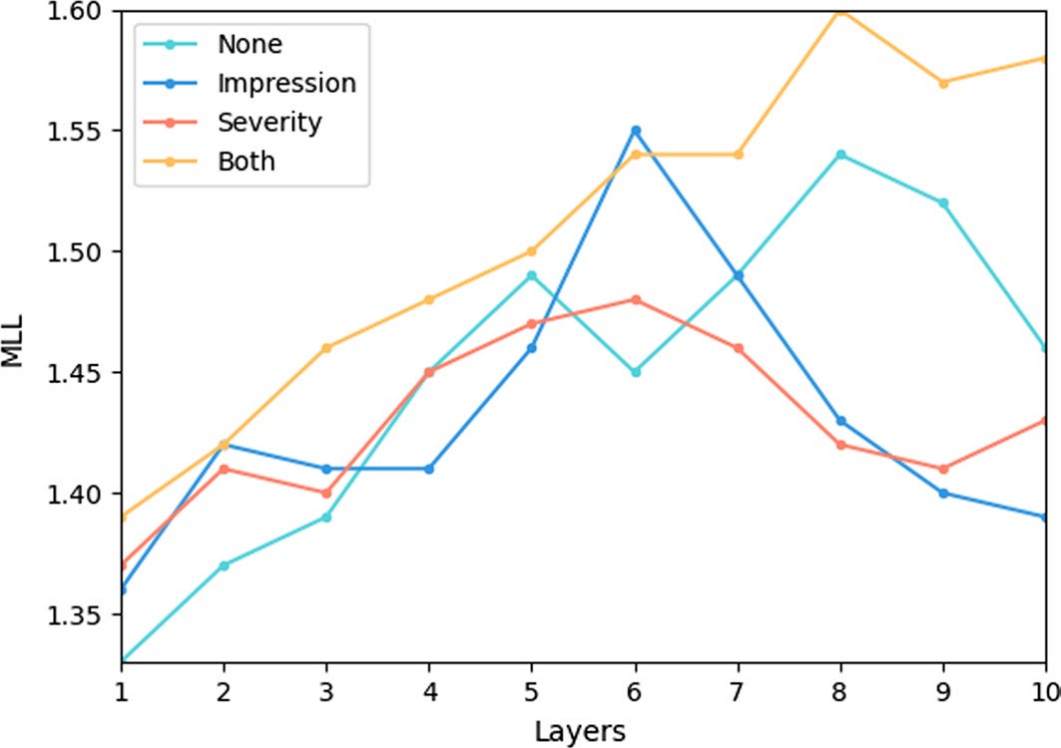
Comparisons of different layers

Figure 6 illustrates the MLLs with different layers on the test set. The trend of results are similar with Fig. 5. When the number of layers increased from 1 to 10, all methods reported increasing performance trends. This shows the model capability is proportional to its fitting ability. The performances of all models decreased after their peak value, which implies that stacking more layers does not extract more useful features, although the number of parameters and running time increase accordingly. Consequently, the model scale should match the dataset, using a large model to fit small datasets might not be a feasible solution. In addition, Multi-object MODE reported superior performances than its original versions. This shows that multi-objective structure can indirectly scale up the dataset and extract more useful features.

#### Influence of template size

**Fig. 7:**
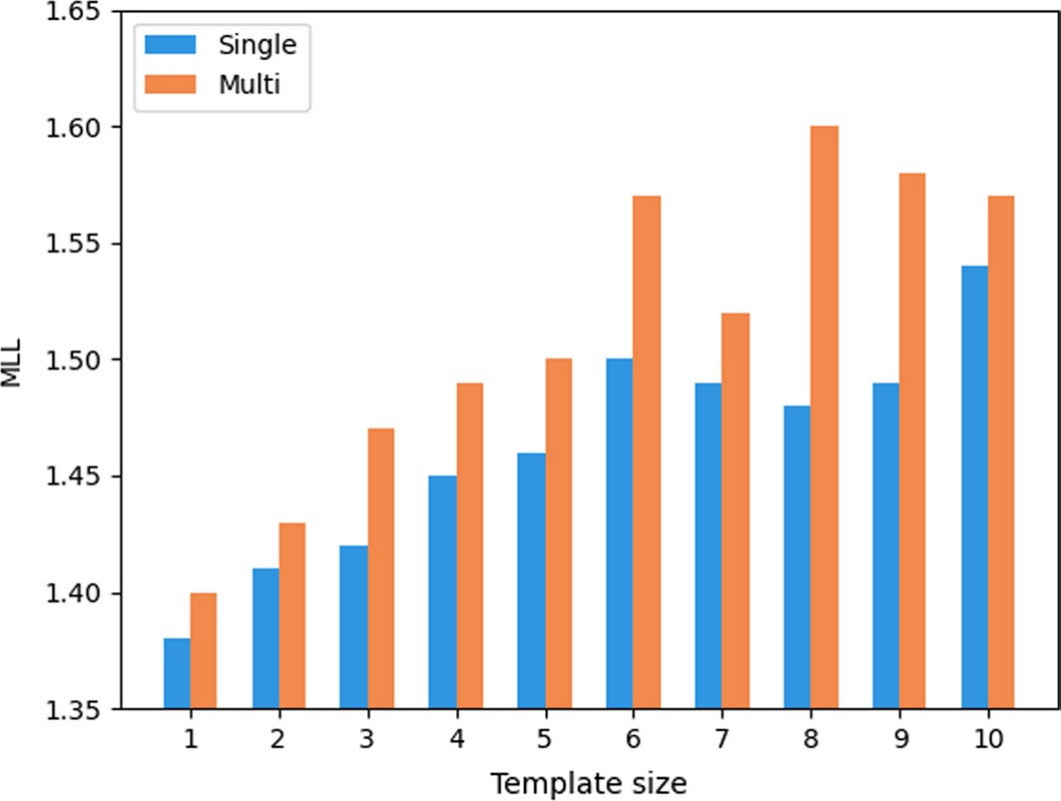
Comparisons of different template settings

Figure 7 illustrates the MLLs with different template settings on the test set. The template size is controlled by three hyper-parameters, *r*, *c* and *k*. The former two parameters affect the hidden size and the last parameter affects the template size. We fix *r* and *c* as 261 and adjust *k* from 1 to 10. In multi-objective training mode, the trend of MLL increases with *k*. This shows the template capability of MODE is coherent with NN models, and larger Template will produce higher performance. To validate whether multi-objective or the template contributes to the improvement, we trained the main task itself, without auxiliary tasks, with the Template. As expected, in single-task mode, MODE reports a relatively inferior performance than its multi-objective version. Consequently, multi-objective can indeed help the model to learn extensive knowledge and solid semantic distribution from auxiliary tasks, and improve the performance of the main task.

### Case study

**Fig. 8:**
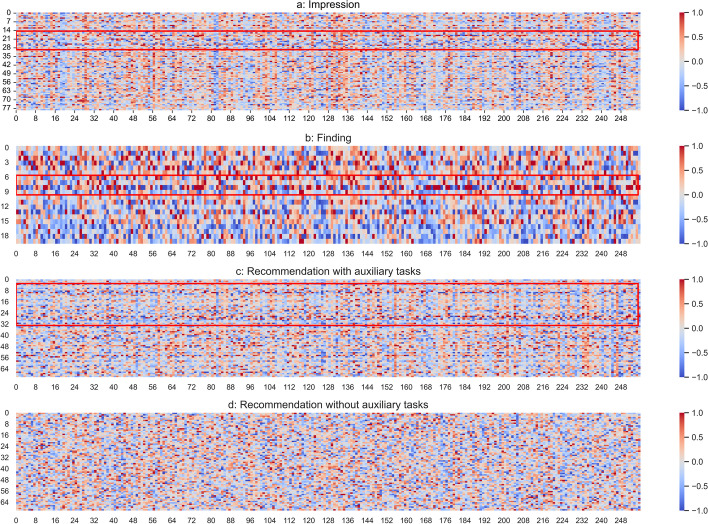
The visualization of an ultrasound report

To further investigate the effectiveness of our method, we selected some cases and visualized their representation matrices. Figure 8 shows the data visualization results of an ultrasound report.

Figure 8a and b are the visualizations of Finding and Impression texts in the Severity task, Fig. 8c and d are the visualizations of predicted recommendations in the ATR task, with and without the help of auxiliary tasks. In each figure, rows and columns represent words and semantic distribution respectively. It is observed in Fig. 8a and b that the representation vectors of some words are obviously different from the others, indicating that these words may have special relations and are critical to analyze the conditions of patients. As shown in Fig. 8c, the ATR task inherits these important words, which will benefit the generation of recommendation. In opposite, without the help of auxiliary tasks, shown in Fig. 8d, the visualization of the predicted recommendation tends to be evenly distributed, indicating that ATR task cannot find informative words and lead to the insufficient performances. Consequently, the learned knowledge of auxiliary tasks can be used to increase the performance of the main task.

### Space cost

**Fig. 9:**
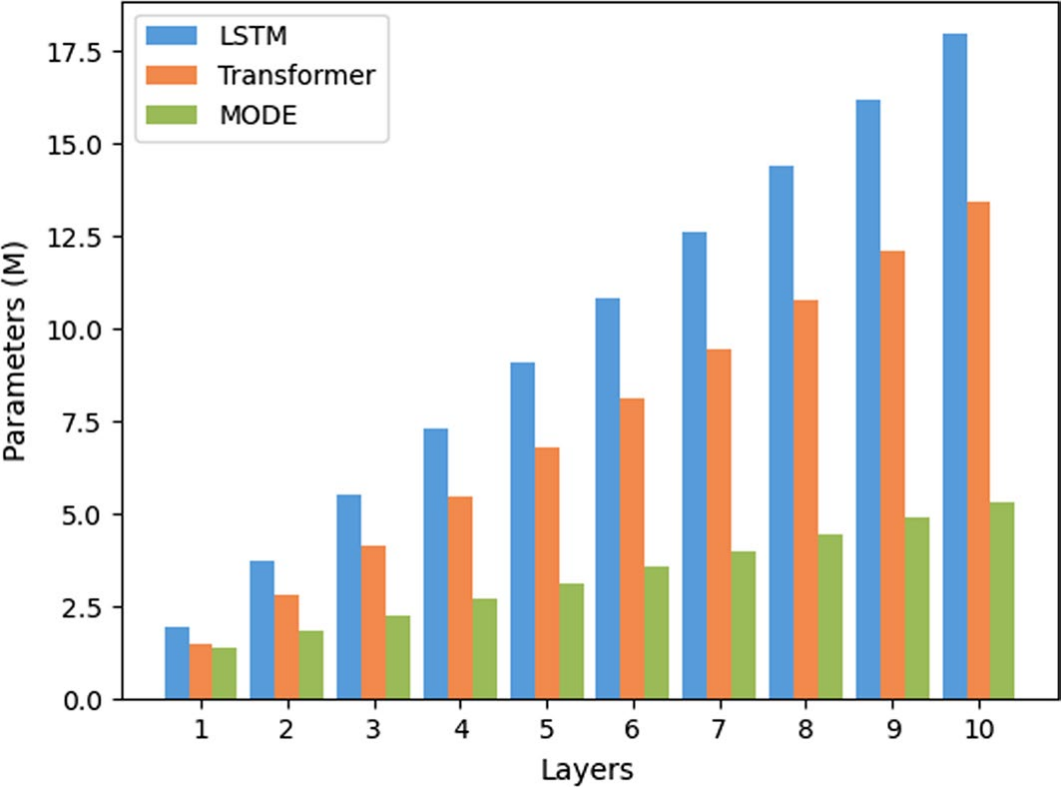
The number of parameters with different number of layers

**Fig. 10:**
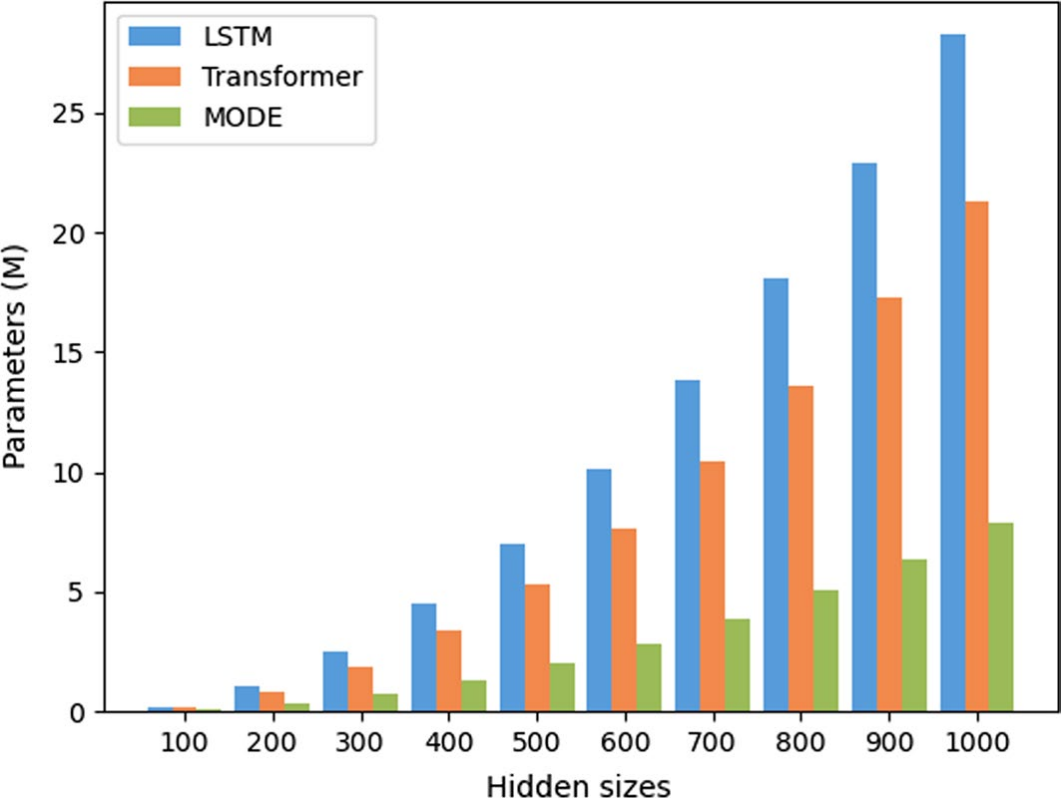
The number of parameters with different hidden sizes

It is intuitive that the space complexity of MODE is larger than existing models since MODE adopts multiple NN models. However, with the help of SPS, MODE has fewer parameters than a Multi-Task method should have. To illustrate this conclusion, we use the parameter size to show the impacts of multiple training objectives on space cost. Figure 9 illustrates the number of parameters of MODE, as well as its competitors, with different layers. In general, more layers will lead to high complexity, and the memory cost grows in proportion to the number of layers. Still, too large memory may introduce redundant and invalid information so as to negatively affect the generation process and lead to over-fitting. In comparison, the number of parameters of MODE grows slower than existing models, for most of the parameters are stored as the Template, and MODE only stores kernels and bias, which has much fewer parameters than the linear modules. It is worth noting that each model of LSTM, CNN and Transformer contains only one task, but MODE contains all of the three tasks. Although MODE has multiple tasks, its parameters are much fewer than existing models and have a relatively flatten increase trend. It is demonstrated that merely small amount of parameters are introduced when adding tasks and models in the memory. This observation suggests that the proposed MODE is effective and efficient in space cost. Similarly, Fig. 10 shows the number of parameters with different hidden sizes, which reaches the same outcome with the situation of different layers.

### Interpretability

**Fig. 11:**
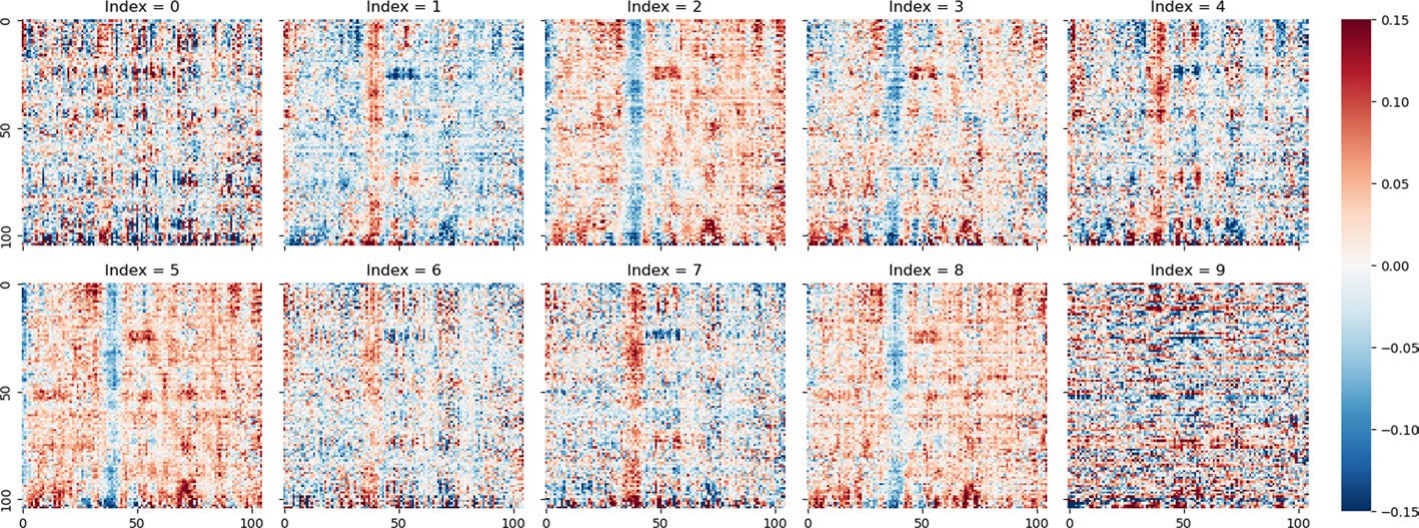
Differences among template slices

We explore the parameter distribution of MODE to study its interpretability. To make clarity representations, we set template size with [10, 105, 105] and the slices of the Template are illustrated in Fig. 11. In the figure, we use heat-maps to visualize the parameters, the red dots represent positive values and blue dots represent negative values. At the first glance, there are three kinds of parameter distributions, in which the 0th and the 9th indices have little distinguishable information, the 1, 4, 6, 7th slices and the remains have similar structures but opposite values. The first and the last slices may store common knowledge or unimportant features, while other slices may store semantic features related to different training objectives. Each slice in the 2–8th slices has two notable vertical lines and a set of distributed clusters, and the second line has the same sign with the corresponding clusters. Obviously, red and blue lines, as well as clusters, indicate that the corresponding slices would like to pay more attention to these areas, which may be the key factors or important context words.

**Fig. 12:**
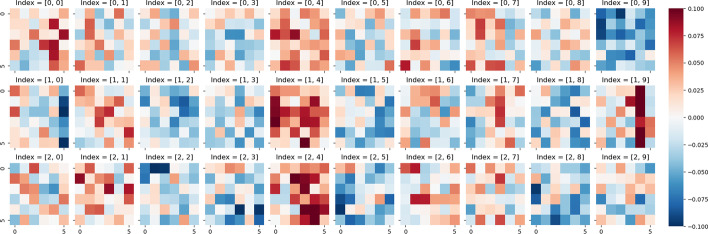
Parameter distribution of SPS kernel

To further explain how MODE utilizes the Template, we present the distribution of SPS kernels. Figure 12 illustrates an example of a SPS kernel which is used to generate parameters for the last Attention module of the Transformer. Corresponding to the Fig. 11, we set the hidden size of Transformer with 100, and the kernel size should be set with [10, 1, 6, 6]. In the figure, the first term of indices $\{0, 1, 2\}$ denote the kernels of ${\varvec{W}}_q$, ${\varvec{W}}_k$ and ${\varvec{W}}_v$ respectively, the second term of indices $\left\{ 0 \sim 9\right\}$ denote the weights of each slice in Fig. 12. At the first glance, we can see that the SPS kernels of ${\varvec{W}}_q$ and ${\varvec{W}}_k$ have relatively large values in the 4 and 9th slices and small values in others. ${\varvec{W}}_v$ has a similar situation but the 4, 5, 8th slices are large (both positive and negative). It can be noting that, in Fig. 11, the vertical line of the 4th slice is positive, which has the same sign of the 4th kernel. This indicates that the 4th slice is strongly enhanced by the kernel. In opposite, although the parameters of the 5th kernel of ${\varvec{W}}_v$ is large, the corresponding kernel of ${\varvec{W}}_q$ has small values, near to zero, indicating that the 5th slice is suppressed by the kernel. The similar situation also happens in other kernels and slices. Consequently, each module will weight specific areas of the Template, and activating different areas of the Template will produce various semantic features.

### Final results

The final results on the test set are shown in Table 3. In addition to training time per epoch, test times are additionally reported. We use the best settings on the development dataset for all models.

**Table 3:** Statistics of the final results

Preference	Time (ms)	tst. MLL	Param (M)
LSTM [19]	875	1.49	14.1
Transformer [22]	545	1.52	9.6
LSTM [20]	993	1.56	14.5
Transformer [26]	568	1.54	10.2
MODE	557	1.60	4.12

As shown in Table 3, the final results on the Ultrasound dataset are consistent with the development results. Internal and external methods have their own merits in handling the ATR task. Specifically, internal methods [19, 22] have fewer parameters and test time while external methods [20, 26] have better performance. The reason is external methods have to learn a large scale of dataset in their training stages, and more complex structures are needed. In comparison, internal methods have relatively small structures since too large models will lead to over-fitting in limited datasets. These results illustrate that the scale of model structure should match the dataset scale, a larger model can extract abundant and highly qualified features but need more training samples. From this viewpoint, many existing methods have tried trade-off, not essential solutions, to solve small-scale and professional datasets.

MODE gives highly competitive results when compared with existing methods in the literature and reports relatively less parameters and test times. To improve the performance, MODE adopted a larger and more complex structure. To fully train MODE, we indirectly scale up the dataset by defining multiple training objectives to learn different aspects of knowledge. Meanwhile, we use SPS to take advantage of learned knowledge and decrease the space cost. Consequently, MODE has the merits of both internal and external methods, and achieved superior performance.

## Conclusion

This paper tackles the contraction between insufficient training samples and professional knowledge in medical datasets. We propose the Multi-Objective Data Enhancement framework for learning and sharing various aspects of knowledge in the limited dataset. Compared with existing methods, MODE has two merits, (1) having the ability to scale up the dataset without external knowledge and (2) concentrating its parameters into a global parameter matrix to decrease the space cost.

The limitation of this paper would be that we only used text data in the experiments. Intuitively, using multi-modal information, such as signal and image data, to train the MODE will achieve better performance. But in applications, there is a quality problem in different data sources. An ultrasound report is a detailed description of a patient’s condition, which is a formal document in clinical diagnosis and has a set of specific writing specifications. In opposite, ultrasound signal or video data are the records of clinical diagnosis, which contains much irrelative information to the report. It is difficult for an NN model to handle such irregular, or even vague data. Considering these two situations, we decided to use text data in the experiments. In addition, the MODE is theoretically a language-insensitive method, and it will function normally in different languages. But we only conducted the experiments in a single-language dataset. The reason is the ultrasound reports were collected and labeled by the cooperative clinicians, which are not familiar with English terms.

In the future work, we will try to utilize multi-modal information and seek for more multilingual datasets.

## Data Availability

The experimental data is available at https://github.com/andrewpark3474/ultrasound-reports.
